# Canine helper-dependent vectors production: implications of Cre activity and co-infection on adenovirus propagation

**DOI:** 10.1038/srep09135

**Published:** 2015-03-16

**Authors:** Paulo Fernandes, Ana I. Almeida, Eric J. Kremer, Paula M. Alves, Ana S. Coroadinha

**Affiliations:** 1 iBET, Instituto de Biologia Experimental e Tecnológica, Oeiras, Portugal; 2 Instituto de Tecnologia Química e Biológica, Universidade Nova de Lisboa, Oeiras, Portugal; 3 Institut de Génétique Moléculaire de Montpellier, CNRS – Universities of Montpellier I and II, Montpellier, France

## Abstract

The importance of Cre recombinase to minimize helper vector (HV) contamination during helper-dependent adenovirus vectors (HDVs) production is well documented. However, Cre recombinase, by inducing DNA double-strand breaks (DSBs), can cause a reduced proliferation and genotoxic effects in cultured cells. In this work, Cre-expressing cell stability, co-infection and their relation to adenovirus amplification/HV contamination were evaluated to develop a production protocol for HD canine adenovirus type 2 (CAV-2) vectors. Long-term Cre expression reduced the capacity of MDCK-E1-Cre cells to produce CAV-2 by 7-fold, although cell growth was maintained. High HDV/HV MOI ratio (5:0.1) led to low HV contamination without compromising HDV yields. Indeed, such MOI ratio was sufficient to reduce HV levels, as these were similar either in MDCK-E1 or MDCK-E1-Cre cells. This raises the possibility of producing HDVs without Cre-expressing cells, which would circumvent the negative effects that this recombinase holds to the production system. Here, we show how Cre and MOI ratio impact adenovirus vectors yields and infectivity, providing key-information to design an improved manufacturing of HDV. Potential mechanisms to explain how Cre is specifically impacting cell productivity without critically compromising its growth are presented.

Adenoviruses have received considerable attention as vectors for gene therapy and their genome has been progressively modified to improve safety and efficacy in therapeutic applications. From vectors with the deletion of the E1 region to helper-dependent vectors (HDVs) with the deletion of all viral genes, an enhanced capacity for a gene therapeutic insertion from ~7 kb to ~36 kb has been achieved^1^. Most replication-defective adenovirus vectors require for manufacturing and replication a cell line that expresses the adenoviral E1 functions in *trans*. However, since HDVs retain only the inverted terminal repeats and the packaging signal, early and late viral functions need to be provided in a synchronized fashion. Standard productions of HDVs are based on co-infection of HDV and a helper vector (HV), which provides all viral proteins in *trans*. During production, both HDV and HV genomes replicate inside the cells, but the encapsidation of the HV genome is minimized, by flanking its packaging signal (ψ) with recognition sequences for a recombinase constitutively expressed by the cells, such as Cre^2^. Therefore, the E1 transcomplementing cell line for HDV production must also express the recombinase^2^,^3^,^4^. Cre/loxP system was the first system described to efficiently reduce HV contaminant, and is still the most common system used for HDV propagation.

The importance of high levels of Cre to avoid HV propagation is unquestionable^5^. In fact, considering that the remaining HV contaminant was due to limited Cre activity levels that permitted HV to escape packaging signal excision^5^, major advances were made to increase the levels of recombinase during HDV production^6^. On the other hand, the effect of Cre expression on producer cell homeostasis has been undervalued. Cre expression can result in a markedly reduced proliferation and genotoxic effects in cultured cells^7^. The aberrant activity on multiple pseudo loxP sites presented in the mammalian genome^8^ induces DNA double-strand breaks (DSBs) or nicks that are converted to DSBs during DNA replication, leading to arrest of cells in G_2_/M phase because of intolerable DNA damage^7^. Furthermore, although sustained low-levels might be supported by cells without significant toxicity, they cause a slow, cumulative increase in recombination and chromosome abnormalities. Therefore, the stability of cells constitutively expressing Cre recombinase is likely to be impaired. While these effects are not substantiated in literature in the scope of adenovirus production, they are considerably described for several cell lines^7^,^9^,^10^,^11^, including HEK 293 cells^9^, traditionally used to propagate adenoviruses. From a bioprocess point-of-view, failure of producer cell line robustness to maintain performance compromises the viral preparations quality, process yields, reproducibility, economics and the regulatory approval.

Previously, we established MDCK-derived cell lines for the production of canine adenovirus type 2 (CAV-2) vectors^12^. In this work, we studied the impact of Cre recombinase in the scope of an optimal production process for HD CAV-2 with MDCK-derived cells in serum-free medium, emphasizing two aspects: cell line stability and infection conditions. Canine adenovirus type 2 (CAV-2) vectors were selected because of their attractive features to bypass the clinical disadvantages of using human adenoviruses while keeping other advantages^13^,^14^, to address fundamental neurobiological questions^15^,^16^,^17^,^18^,^19^ and to develop potential treatment of neurodegenerative and ocular disorders^20^,^21^,^22^. Moreover, MDCK cells are already approved by the regulatory authorities for the manufacture of vaccines and thus represent a suitable cell substrate that might facilitate the approval of clinical grade CAV-2 vectors production^23^. MDCK-E1-Cre cell line stability was addressed by evaluating growth, Cre activity levels and virus production in cells with increasing culture passages. Different HDV/HV MOI ratios were assayed to determine the best infection conditions to amplify HD CAV-2 and to understand the relation between Cre expression, infection conditions and HV propagation. Here, we show how Cre expression impacts cell performance and adenovirus propagation and how infection conditions significantly contribute to reduce HV contamination during HDV production even in the absence of Cre-recombinase.

## Results

MDCK-E1-Cre cells, once adapted to grow in Optipro SFM, were maintained in culture for 20 more passages (P) in duplicates, with and without antibiotic pressure, and frozen at P0, P10 and P20 (see material and methods section). MDCK-E1 cells, subjected to same procedure, were used as a control for CAV-2 vector production. The experiments to determine the best infection conditions for HD CAVGFP (HDV) production were performed with cells at P0.

### Cell line evaluation at different culture passages

#### Growth characteristics of MDCK-E1-Cre

**Figure 1:**
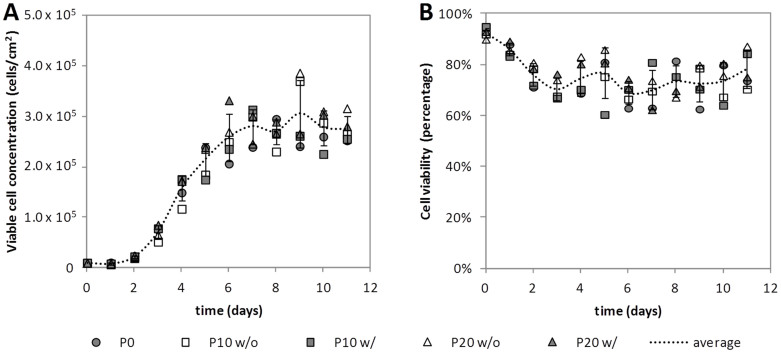
Effect of cell culture passage and antibiotic pressure on MDCK-E1-Cre cell growth (A) and viability (B). Dashed line represents the average of data points and error bars the corresponding standard-deviation. MDCK-E1-Cre cell growth was similar to MDCK-E1 cells^23^; for a matter of simplicity data points for MDCK-E1 cells are not shown. w/: with antibiotic pressure; w/o: without antibiotic pressure.

Growth curves of MDCK-E1-Cre cells obtained in the different cell culture passages were performed in 25 cm^2^ t-flasks. Concentration and viability were determined every day (Figure 1).

The results show that cell growth was similar in all culture passages, regardless of antibiotic pressure. On average, MDCK-E1-Cre cells showed a specific growth rate of 1.00 ± 0.07 day^−1^ (Figure 1A) and a minimum cell viability of 63 ± 3% obtained during stationary phase (Figure 1B). These results indicate that increasing passages and selective pressure had no impact on MDCK-E1-Cre cell growth.

#### Cre expression and excision efficiency of MDCK-E1-Cre

**Figure 2:**
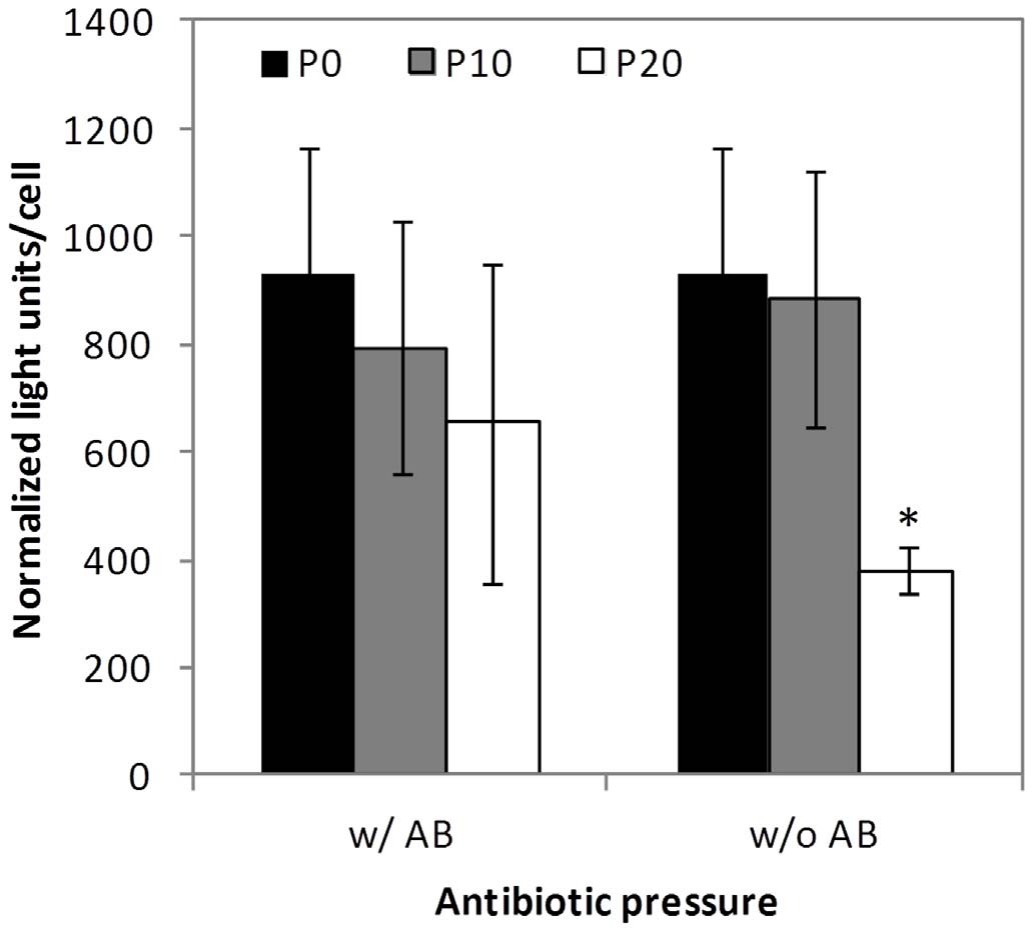
Effect of cell culture passage and antibiotic pressure on Cre-activity of MDCK-E1-Cre cells. Light units were normalized to cell concentration and background obtained with MDCK-E1 cells. Values are shown as average ± standard-deviation (n = 3). w/AB: with antibiotic pressure; w/o AB: without antibiotic pressure. * *p* = 0.01, given by a single factor Anova analysis against the corresponding P0 values.

To evaluate the impact of increasing passages in Cre expression, two approaches were used. The first estimates Cre activity through the level of luciferase activated by the excision of a stop sequence flanked by loxP sites (view materials and methods section) (Figure 2). In the second approach, Cre activity was estimated by evaluating the reduction in HV JBΔ5 propagation (due to excision of packaging genome) in MDCK-E1-Cre cells (Table 1). The results obtained from both methods were similar. A slight decrease in Cre activity was obtained in cells at P20 when maintaining the antibiotic pressure (Figure 2), nevertheless this reduction was not statistically significant nor significantly altered the HV excision efficiency (Table 1). Without the selective pressure, a further 2.5 ± 0.5 fold decrease in Cre activity was observed from P10 to P20 (Figure 2), which contributed to approximately 3-fold reduction in the capacity for HV genomes excision (Table 1).

#### CAV-2 production of MDCK-E1-Cre

To evaluate the impact of increasing subculture passages on the capacity to produce viral vectors, production assays with CAVGFP (ΔE1) were performed. MDCK-E1 cells maintained in parallel under same passages number were added to the experiments and used as controls for ΔE1 production.

**Figure 3:**
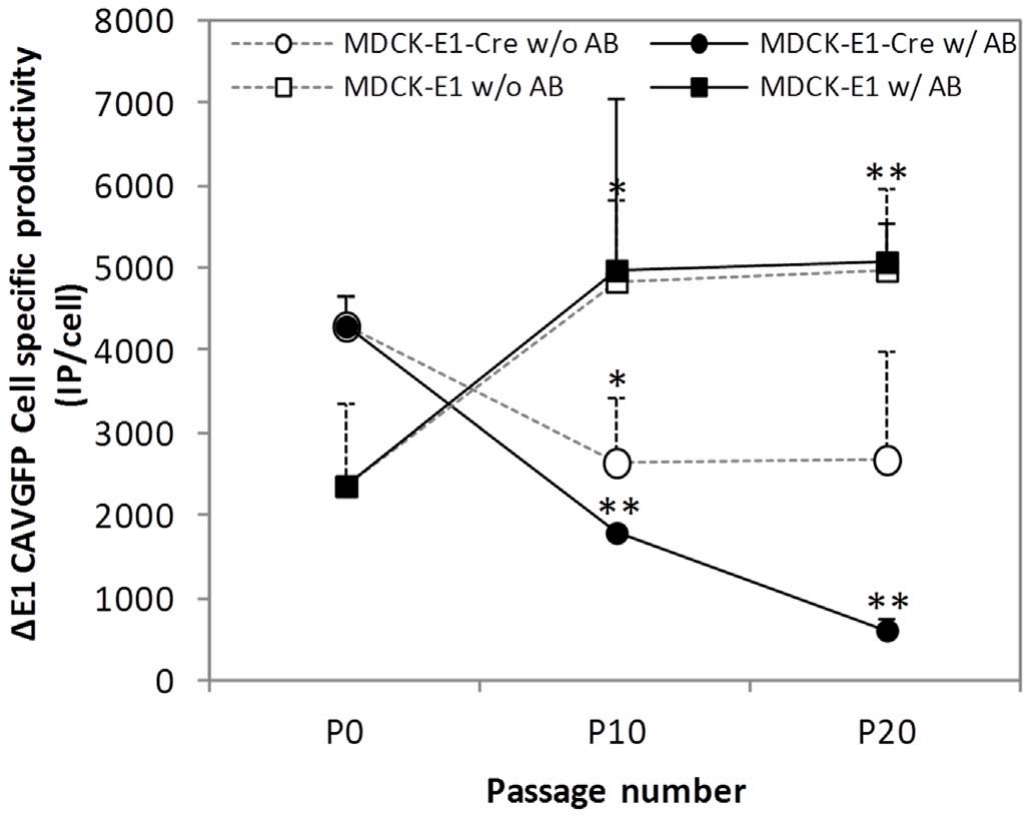
Effect of cell culture passage and antibiotic pressure on MDCK-E1-Cre cells specific productivity. As a control, MDCK-E1 cells under same conditions were added to the experiments. Values are shown as average ± standard-deviation (n = 3). w/: with antibiotic pressure; w/o: without antibiotic pressure. * *p* < 0.05, ** *p* < 0.005, given by a single factor Anova analysis against the corresponding P0 values.

In MDCK-E1 cells, an increase of 2.1 ± 0.3 fold in productivity was observed with increasing cell passages and regardless of selective pressure (Figure 3). Such feature has been reported for these cells^12^. On the other hand, MDCK-E1-Cre cells showed a decrease in the production of ΔE1. A slight reduction of 1.6 ± 0.5 fold was observed in cells without selective pressure at P10, in which productivity was then maintained up to P20. On the other hand, MDCK-E1-Cre under selective pressure showed a consecutive reduction in ΔE1 production capacity with increasing passages, achieving a 7.1 ± 1.7 fold-decrease at P20. These observations indicate that production capacity of cells was hampered by maintaining Cre activity through selective pressure.

### Establishing the best conditions for HD CAV-2 production

To exclude any cell passage effect, the following experiments were performed in MDCK-E1-Cre and MDCK-E1 cells at P0.

#### HDV/HV MOI ratio

To establish optimal conditions to produce HDV (maximum productivity and lowest HV contamination), production assays under different MOI ratios (5 to 1, 5 to 0.5, and 5 to 0.1 IP) were performed. MOI ratio of 5 to 5 was initially tested, but critically compromised cell viability and production of either HDV or HV (data not shown).

**Figure 4:**
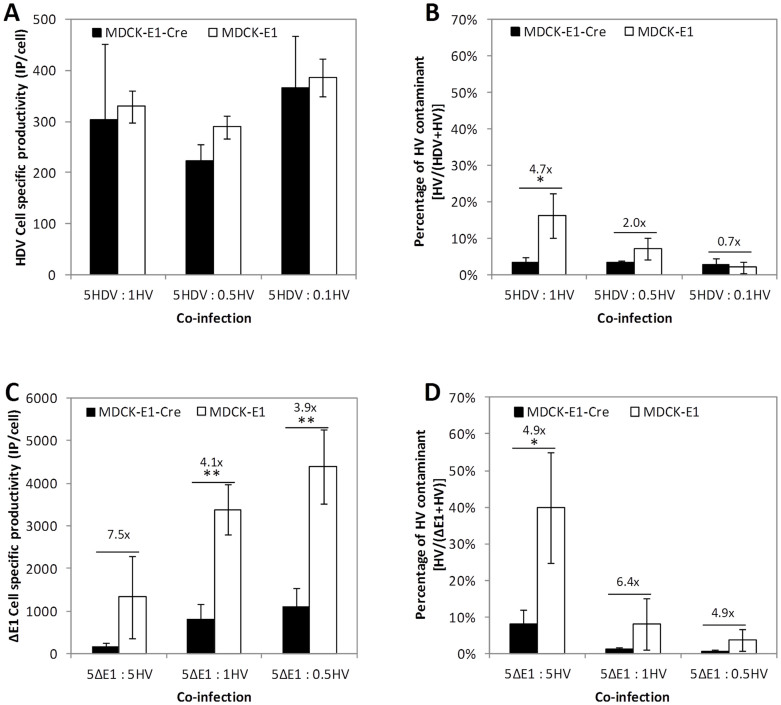
Effect of MOI ratio on virus production and HV contaminant using MDCK-E1-Cre and MDCK-E1 cells. (A, B) Cell specific productivity of HDV (A) and resulting HV contaminant (B). (C, D) Cell specific productivity of ΔE1 (C) and resulting HV contaminant (D). Values are shown as average ± standard-deviation (n = 3). * *p* < 0.05, ** *p* < 0.005, given by a single factor Anova analysis.

Similar HDV specific productivities of approximately 300 IP/cell were obtained using the different MOI ratios tested (Figure 4A). Even with a MOI as low as 0.1, HDV productivity was still maintained. When using MDCK-E1-Cre cells, HV contamination levels were similar in the three co-infections (2–3%) (Figure 4B).

To further understand how Cre recombinase impacts HV propagation in the different MOI ratios, the assays were also performed in MDCK-E1 cells (Figure 4A, B). The results show a similar HDV production in both cells (Figure 4A), however a clear proportion between HV MOI and its contaminant levels was observed (Figure 4B). While at MOI 1 of HV a high contamination was observed (16.3%), a decrease in HV contaminant to 2.0% was possible when decreasing MOI of this vector. Moreover, contaminant levels of HV with MDCK-E1 cells in 5HDV: 0.1HV co-infections were similar to those obtained with MDCK-E1-Cre cells. These results indicate that co-infection with the lowest HV MOI tested herein, i.e. 0.1 of HV, was sufficient to minimize contamination during HDV production.

#### The impact of MOI ratio in co-infections

Given that HDV production requires co-infection with HV, the impact of co-infection conditions on adenovirus propagation and HV contaminant levels were further analyzed using other CAV-2 vector. Infections assays under different MOI ratios were reproduced by replacing HDV for ΔE1 CAV-2 vectors. Similarly to HDV assays, cells were co-infected with ΔE1 (MOI 5) and HV at MOI 5, MOI 1 or MOI 0.5 using MDCK-E1 and MDCK-E1-Cre cell-lines.

The production of ΔE1 vector increased when HV MOI was decreased for both cell lines (Figure 4C). However, MDCK-E1-Cre cells showed lower capacity to produce CAVGFP than MDCK-E1 cells, especially under high virus input. Similarly to the assays with HDV, the proportion of HV was minimal in MDCK-E1-Cre cells, and highly dependent on the MOI when MDCK-E1 cells were used (Figure 4D). However, in each infection condition assayed, a constant fold-reduction (5- to 6-fold) in HV contamination levels was observed when comparing MDCK-E1 to MDCK-E1-Cre cells. These results corroborate that the MOI ratio used to infect cells determines the proportion of each vector in the final production pool, i.e. which vector and its propagation is favored.

#### The role of Cre recombinase and MOI on the propagation of HV

**Figure 5:**
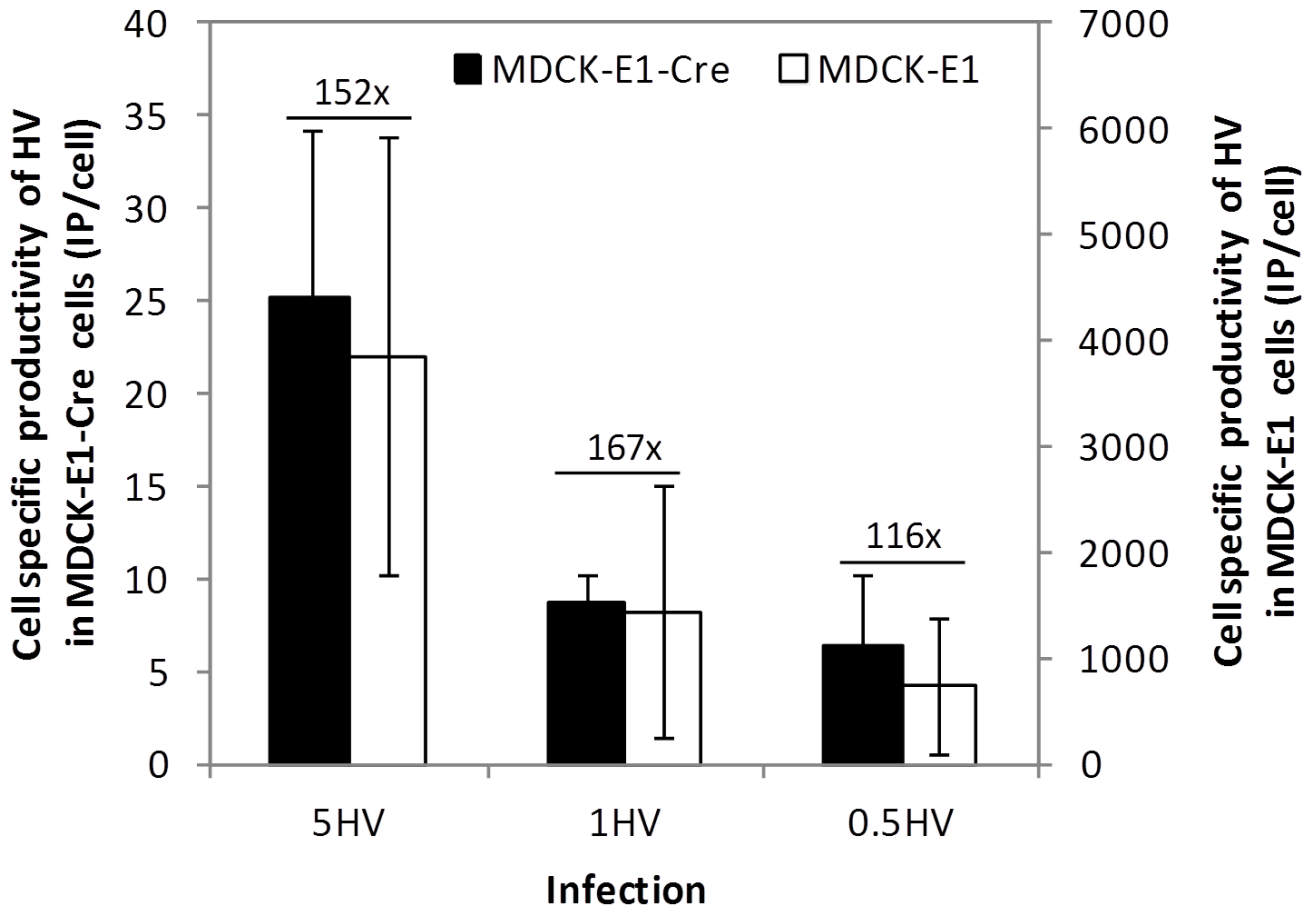
Effect of MOI on HV production in MDCK-E1-Cre and MDCK-E1 cells. Values are shown as average ± standard-deviation (n = 3).

To gather a better understanding of the factor behind a low HV contamination in HDV production, the role of Cre recombinase and MOI were evaluated from an HV propagation point-of-view. To understand the relation between Cre recombinase and HV propagation under different MOIs, infections with HV using MOI 5, 1 and 0.5 were assayed in MDCK-E1-Cre and compared to those obtained with MDCK-E1 cells (Figure 5). A decrease in HV production was observed in MDCK-E1-Cre cells in comparison to MDCK-E1 cells due to excision of the packaging signal by Cre activity. Cell specific productivity was proportional to the MOI used in both cell lines. Furthermore, the differences in IP/cell of MDCK-E1-Cre versus MDCK-E1 cells in the different MOIs tested were shown to be similar, following on average a fold-reduction of 145 ± 23. These observations indicate that the number of HVs escaping from excision was proportional to the MOI used.

#### The relation between viral particles infectivity, Cre and packaging

Physical (genome-containing) particles of HDV, ΔE1 and HV were quantified and normalized to the infectious titer to analyze the infectivity in the different conditions assayed.

**Figure 6:**
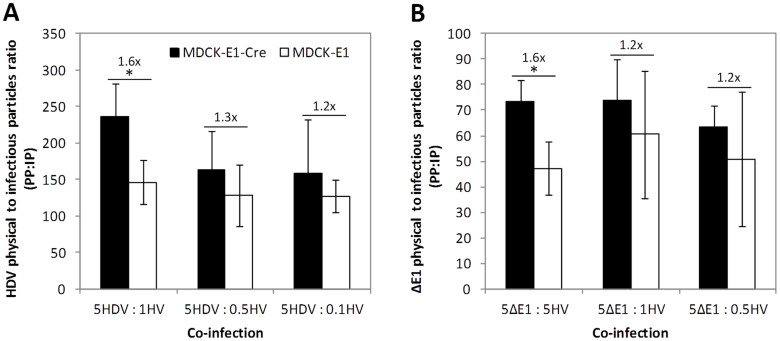
Comparison of physical to infectious particles ratio for HDV (A) and ΔE1 (B) obtained with MDCK-E1 and MDCK-E1-Cre cells in the different co-infections. Values are shown as average ± standard-deviation (n = 3). * *p* < 0.05, given by a single factor Anova analysis.

The results show a higher PP:IP ratio for HDVs than for ΔE1 (Figure 6), as previously described^24^. When using MDCK-E1 as producer cells, the PP:IP ratios of HDV and ΔE1 were similar along the different co-infections (Figure 6A, B). However, HDV and ΔE1 PP:IP ratios obtained with MDCK-E1-Cre cells were consistently higher than those obtained with MDCK-E1 cells when co-infecting cells with high MOI of HV. Together, these observations show that MDCK-E1-Cre cells generate more non-infectious particles when using high MOI of virus.

When evaluating HV, no significant differences in PP:IP ratios were observed between the different MOIs or cell lines used (Table 2), probably because of the high interassay variability obtained when titrating infectious HV.

## Discussion

While helper-dependent vectors (HDVs) continue to demonstrate a high therapeutic potential, the production standardization of these vectors is still limiting their wide availability in gene delivery assays and trials. In this study, we evaluated Cre-expressing cells stability, infection conditions and their relation to adenovirus amplification and helper vector (HV) contamination in serum-free medium conditions to establish a reproducible production process for HD canine adenovirus type 2 (CAV-2) vectors. We found that constitutive Cre expression affects the long-term capacity of cells to produce adenoviruses, and co-infection conditions using low HV MOI can effectively reduce HV contamination even when using cells without Cre recombinase.

HDV producer cells need to be in culture for longer periods of time as cells are needed for serial amplifications until sufficient vectors are generated^2^,^25^,^26^,^27^. On the other hand, the expression of Cre recombinase, due to its toxicity^7^, is likely to impair cell line performance. Therefore, the evaluation of producer cell line stability in the scope of HDV production becomes critical. The consistent MDCK-E1-Cre cells growth profile under increasing subculture passages (Figure 1) indicates that Cre expression was supported by the cell line, probably because of non-toxic levels and/or the cells capacity to correct Cre-induced DNA double-strand breaks (DSBs)^7^. However, when evaluating virus production, a consecutive reduction in ΔE1 vectors (CAVGFP) productivity along culture passages was observed (Figure 3), namely in the cells forced to express Cre recombinase through selective pressure from P10 to P20 (Figure 2). MDCK-E1-Cre cells in Optipro SFM medium can be thus used for virus production during 10 culture passages after thawing. The factor(s) and/or mechanism(s) behind these observations must be such that despite impacting virus replication, cell homeostasis is balanced enough to sustain growth under the presence of Cre. Furthermore, any effect of E1 expression levels on CAV-2 production should be ruled out, since E1 levels between MDCK-E1-Cre and MDCK-E1 cells in same conditions (passage number and antibiotic pressure) were found to be similar (data not shown). The 116 cell line, one of the several Cre-expressing cell lines derived from HEK 293^28^ and established by Palmer and Ng^26^, was able to keep adenovirus productivities up to 86 passages when maintained under selective pressure. While these results differ from ours, the evaluation of other Cre-expressing cells established for HDV production^28^ should be pursued to understand how broad/specific our results with MDCK-based cells are and how host cell/cell clone can also contribute to the impact that Cre has on the resulting cell performance.

The best infection conditions for HDV production must be well established as the MOI ratio of HDV/HV impacts the contamination levels of HV in co-infections. Similarly to what was shown with human HDV production^29^, the best strategy to produce HD CAV-2 was under co-infections of 5 HDV: 0.1 HV, where maximum HDV productivity were ensured and HV contamination the lowest (Figure 4). Indeed, a low HV MOI can benefit HDV production by reducing the levels of excised helper DNA molecules after Cre recombinase activity, that are known to contribute to recombination between viral vectors^30^, and circumventing the need to develop alternative strategies to attain higher levels of Cre than those supported by the cell line^6^. The relation between MOI ratio and HV contamination levels, namely that obtained in co-infections using MDCK-E1 cells (Figure 4B, D), indicates that MOI ratio is an effective strategy to unbalance the competition for amplification of HV in relation to HDV or ΔE1. In fact, this led to contamination levels as low as those obtained with MDCK-E1-Cre cells when producing HDV (Figure 4B), raising the possibility of producing HDV without the need of a recombinase when optimal MOI ratios are used. Therefore, as soon as HDV titers permit the establishment of such MOI ratio (usually after rescue step and 1^st^ amplification), the subsequent amplifications can be moved to cell lines without Cre. This would lead to better vector yields, surpassing the negative effects that Cre has on cell performance^7^, recombination between vectors genomes^30^ and virus amplification (Figure 3).

The analysis of PP:IP ratios also indicated that more non-infectious particles were generated when using MDCK-E1-Cre cells as producer cells, especially when co-infections were performed with high MOI of HV (Figure 6). Although the differences in PP:IP ratio between MDCK-E1 and MDCK-E1-Cre cells were rather low (up to 1.6-fold differences), they were statistically significant under high MOI of HV. More importantly, given that the PP:IP ratio of clinical grade adenovirus vector is limited by the Food and Drug Administration, special attention must be paid to this. These findings substantiate the need to minimize the use of Cre recombinase (expressing cells) and lower HV MOI to maximize HDV quality. Following these observations, cell physiological state imposed by the expression of Cre recombinase, probably aggravated under high HV MOI, may be compromising the success of virus replication cycle and virus particle maturation process. The relation between Cre activity, infection conditions and virus maturation should be thus further clarified to understand the mechanism behind the generation of less infectious particles.

Apart of generating more non-infectious particles under high virus MOI (Figure 6) and reducing virus production at high passage number (Figure 3), MDCK-E1-Cre cells also impacted CAV-2 production when co-infected with HV. In fact, when comparing the specific productivity of MDCK-E1 and MDCK-E1-Cre cells in ΔE1 + HV co-infections, a reduction in ΔE1 amplification was observed with MDCK-E1-Cre cells, specially under high HV MOI (Figure 4C). This feature is specific to co-infections with HV in MDCK-E1-Cre cells as this was not observed after infecting MDCK-E1-Cre and MDCK-E1 with increasing MOIs (from 5 to 40) of ΔE1 alone (Figure 3 and data not shown). Since the difference between co-infections with MDCK-E1-Cre and MDCK-E1 cells is the occurrence of HV genomes excision, these observations substantiate a relation between high number of excised HV genomes and inhibition of ΔE1 amplification.

To support Cre recombinase activity, cells must present a consistent DSBs repair system. Such mechanism is involved in the inhibition of adenovirus replication: viral genomes are sensed as DSBs, activating DNA damage response and the repair mechanism concatenate viral genomes making them unavailable for the subsequent replication process^31^. Adenoviral E4 11 k (E4orf3) or the complex of E4 34 k (E4orf6) and E1B proteins counteract and are sufficient to inhibit DSBs repair system, thereby preventing genome concatenation^32^,^33^,^34^,^35^. Given the link of Cre – DSBs repair – virus amplification, we hypothesize that the reduction in CAV-2 production could be due to an up regulation of DSBs repair mechanism. Assuming that Cre-expressing cells should have an up regulated DSBs repair system, MDCK-E1-Cre cells are likely to be more prone in sensing viral DNA as DSBs than MDCK-E1 cells. If MDCK-E1-Cre cells are challenged with high MOIs of HV, more DNA breaks are exposed as more viral genomes are being excised, which might result in a higher activation of DSBs repair system, explaining the inhibition of ΔE1 propagation under high HV MOIs (Figure 4C). Accordingly, MDCK-E1-Cre cells with increased DSBs repair system, being more prone to repair Cre-induced damages, might dominate cell population during culture passages due to proliferative advantage. Therefore, the DSBs repair system of cells maintained in culture for long periods would be consistently more active and sensitive to viral genomes, which might explain the subsequent reduction of ΔE1 production in MDCK-E1-Cre cells along passages (Figure 3). A model where DSB repair system interfere with the typical adenovirus propagation must fit with a scenario which E4 levels attained during infection are no longer enough to inhibit this mechanism^32^,^33^,^34^. In addition, the occurrence of genome concatenation should be observed to confirm these assumptions^31^.

In conclusion, despite similar growth profile of MDCK-E1-Cre cells with increasing passages, the long-term Cre recombinase expression reduced their capacity to produce adenoviruses. Not only that, but more non-infectious particles were generated with these cells (up to 1.6 fold-increase) in HV co-infections under high MOIs. The best conditions to produce HDV followed a high HDV/HV MOI ratio with a HV MOI as low as 0.1. This favored HDV propagation and infectivity and limited the generation of contaminant HV. In fact, such MOI ratio was capable of attaining low HV contamination levels, with or without the activity of Cre, raising the possibility of producing HDVs without Cre-expressing cells when optimal infection conditions are used. This work unveils the impact of the Cre recombinase system and MOI ratio on adenovirus propagation, highlighting important bioprocess parameters that must be considered to design an improved manufacturing of HDVs.

## Methods

### Cell lines and culture media

MDCK-E1 cells^12^ are MDCK (ECCAC 841211903) derived and stably express the E1 region from CAV-2 and the neomycin resistance gene. MDCK-E1-Cre cells^12^, in addition to E1, also express Cre recombinase and zeocin resistance gene. MDCK-E1 and MDCK-E1-Cre were sequentially adapted to Optipro serum-free medium (Gibco, Paisley, UK) with 4 mM glutamax (Gibco) by increasing the percentage of Optipro medium in subculture passages. Cells were subcultured twice a week in 150 cm^2^ t-flasks and maintained in an incubator with humidified atmosphere of 5% CO_2_ in air at 37°C. For splitting or collecting cells, the monolayer was washed with PBS, incubated with 0.25% (w/v) trypsin-EDTA (Gibco) at 37°C until detachment started to become evident (up to 15 min) and cells suspended in culture medium. Cells were then pelleted at 300 *g* during 10 min and re-suspended in fresh medium. To evaluate the effect of culture passages, cells were maintained in duplicates, either with or without antibiotic pressure. For antibiotic pressure, 500 μg/mL of Geneticin (G418) (Invivogen, Toulouse, France) was added to culture medium for MDCK-E1 cells maintenance, while for MDCK-E1-Cre cells G418 plus 500 μg/mL of Zeocin (Invivogen) was used. Cells were frozen once adapted to Optipro medium (P0) and after 10 and 20 cell culture passages in CryoStor CS10 (Sigma-Aldrich, St. Louis, MO, USA) using a Mr. Frosty Freezing Container (Thermo-scientific, Rockford, IL, USA).

### Viral vectors

CAVGFP, JBΔ5 and HD CAVGFP are vectors derived from CAV-2 strain Toronto A 26/61, GenBank J04368. CAVGFP^13^ and HD CAVGFP^16^ are E1-deleted (ΔE1) and HD vectors, respectively, and contain an eGFP expression cassette. JBΔ5 is a helper vector (HV) containing loxP sites flanking the packaging domain and a RSV-*lacZ* expression cassette^16^. CAVGFP, JBΔ5 and HD CAVGFP viral stocks were prepared and purified by CsCl gradients as described previously^12^,^13^.

### Cell growth assays

MDCK-E1-Cre growth assays in static cultures were performed using an inoculum of 1 × 10^4^ cells/cm^2^ in 25 cm^2^ t-flasks with a working volume of 10 mL. Cell concentration and viability was determined by the trypan blue exclusion method using a 0.1% (v/v) solution prepared in PBS and counting cells in a Fuchs-Rosenthal haemacytometer (Brand, Wertheim, Germany).

### Cre activity

Relative Cre activity was assayed by the level of luciferase activity generated by the adenovirus vector AdMA19 (a gift from F. Graham) after infecting cells^36^. AdMA19 contains the luciferase cDNA under control of the human cytomegalovirus promoter but separated from it by an extraneous spacer sequence composed by a series of initiation and stop codons in several reading frames. This translational “start/stop sequence”, flanked by loxP, disrupts translation. If the cell clone expresses Cre, the stop sequence is floxed out and translation proceeds leading to luciferase expression. Briefly, cells were seeded in 24 well-plates in quadruplicates and infected the day after with 20 IP/cell of AdM19 with medium exchange. Twenty-four hpi, cells were counted and lysed to release luciferase, both in duplicates. The resulting supernatant was collected and the light units were quantified, with a Modulus Luminometer from Turner Biosystems (Sunnyvale, CA, USA) after adding luciferin, and normalized to cell concentration.

### Viral vectors production assays

Cells were seeded at 3 × 10^4^ cells/cm^2^, infected the day after with medium exchange. To evaluate cells productivity at different passage number, infections of CAVGFP at MOI 5 were performed. Excision efficiency was estimated by determining the fold-reduction in helper JBΔ5 production with MDCK-E1-Cre cells infected at MOI 5. The assays for HD CAVGFP production optimization were performed by co-infecting cells with HD CAVGFP at MOI 5 and JBΔ5 at MOI 1, 0.5 and 0.1. To further understand how Cre recombinase and co-infection determine optimal HD CAVGFP production, co-infections of CAVGFP (MOI 5) with JBΔ5 (MOI 5, 1 and 0.5), and single infections of JBΔ5 (MOI 5, 1 and 0.5) were also performed. Moreover, all the infection assays were performed in MDCK-E1-Cre and MDCK-E1 cells. Once infected, cells were incubated for further 48 h and viral samples collected using triton (Sigma-Aldrich) 0.1% (v/v) in Tris-HCl, clarified at 3000 *g* for 10 min at 4°C and stored at −85°C until further analysis.

### Infectious vectors titration

Quantification of infectious CAVGFP and HD CAVGFP was performed by monitoring the expression of GFP, while titration of infectious JBΔ5 vectors was based in lacZ gene expression and β-galactosidase activity as described previously^12^.

### Quantification of physical particles

Viral genomes were extracted and purified by High Pure Viral Nucleic Acid Kit (Roche Diagnostics, Penzberg, Germany). SYBR Green I dye chemistry was used to detect PCR products using Lightcycler system. Previously purified and quantified plasmids coding for E1-deleted CAV-2 genome, GFP or lacZ were used as standards. To ensure the removal of free viral genomes from samples and accurately quantify viral physical particles, a benzonase treatment was performed prior to DNA extraction by adding 2 μl of benzonase (250 U/μl) (Merck Millipore, Darmstadt, Germany) to the same sample volume and incubating for 1 h at 37°C. Benzonase was inhibited during the proteinase K step of DNA extraction protocol. To estimate the number of viral genomes, primers against the expression cassette of each viral vector were used. Primers for GFP gene: forward 5′-CAGAAGAACGGCATCAAGGT-3′ and reverse 5′-CTGGGTGCTCAGGTAGTGG-3′; primers for lacZ gene: forward 5′-ACTATCCCGACCGCCTTACT-3′ and reverse 5′-TAGCGGCTGATGTTGAACTG-3′.

## Author Contributions

P.F. designed the experiments; P.F. and A.I.A. performed the experiments; P.F. analyzed the data; P.F. and A.S.C. wrote the manuscript; E.J.K., P.M.A. and A.S.C. contributed with materials, equipments and analysis tools; all authors discussed the results and reviewed the manuscript.

## Figures and Tables

**Table 1:** Effect of cell culture passage and antibiotic pressure on excision efficiency of HV genomes in MDCK-E1- Cre cells

	Excised/unpackaged HV genomes (%)		
Passages	w/AB		w/o AB
P0		98.9 ± 0.6	
P10	98.4 ± 1.5		97.7 ± 2.3
P20	92.4 ± 7.2		28.5 ± 2.3

Values are shown as average ± standard-deviation (n = 2). Since MDCK-E1 and MDCK-E1-Cre cells have similar productivities at P0 (Figure 3), the HV amplification reduction obtained in MDCK-E1-Cre cells corresponds to Cre-mediated excision of HV genomes avoiding their packaging. The percentage of excised HV genomes was estimated assuming that the differences of HV amplification titers between (a) MDCK-E1 and (b) MDCK-E1-Cre cells corresponds to excised/unpackaged HV genomes as follows: ([a − b]/a). Value (b) was also normalized to the fold-reduction of MDCK-E1-Cre cells productivity observed during passages (Figure 3). w/AB: with antibiotic pressure; w/o AB: without antibiotic pressure.

**Table 2:** Physical to infectious particles ratio of HV obtained with MDCK-E1-Cre and MDCK-E1 cells under the different co-infections

		MOI of HV			
Co-infection with	Cells	5 HV	1HV	0.5 HV	0.1HV
HDV	MDCK-E1-Cre	n.d.	86 ± 12	93 ± 17	85 ± 16
	MDCK-E1	n.d.	75 ± 18	102 ± 33	93 ± 16
ΔE1	MDCK-E1-Cre	58 ± 8	50 ± 9	42 ± 3	n.d.
	MDCK-E1	37 ± 19	38 ± 15	37 ± 7	n.d.

Values are shown as average ± standard-deviation (n = 3); No statistical mean (*p* value < 0.05) was obtained when comparing values of MDCK-E1-Cre with MDCK-E1 cells under same co-infection conditions. n.d.: not determined.
